# Microfluidic assay of circulating endothelial cells in coronary artery disease patients with angina pectoris

**DOI:** 10.1371/journal.pone.0181249

**Published:** 2017-07-13

**Authors:** Shuiyu Chen, Yukun Sun, Kuang Hong Neoh, Anqi Chen, Weiju Li, Xiaorui Yang, Ray P. S. Han

**Affiliations:** 1 College of Engineering, Peking University, Beijing, China; 2 Peking University Hospital, Beijing, China; The Ohio State University, UNITED STATES

## Abstract

**Background:**

Circulating endothelial cells (CECs) are widely reported as a promising biomarker of endothelial damage/dysfunction in coronary artery disease (CAD). The two popular methods of CEC quantification include the use of immunomagnetic beads separation (IB) and flow cytometry analysis (FC); however, they suffer from two main shortcomings that affect their diagnostic and prognostic responses: non-specific bindings of magnetic beads to non-target cells and a high degree of variability in rare cell identification, respectively. We designed a microfluidic chip with spatially staggered micropillars for the efficient harvesting of CECs with intact cellular morphology in an attempt to revisit the diagnostic goal of CEC counts in CAD patients with angina pectoris.

**Methods:**

A label-free microfluidic assay that involved an in-situ enumeration and immunofluorescent identification (DAPI^+^/CD146^+^/VEGFR1^+^/CD45^-^) of CECs was carried out to assess the CEC count in human peripheral blood samples. A total of 55 CAD patients with angina pectoris [16 with chronic stable angina (CSA) and 39 with unstable angina (UA)], together with 15 heathy controls (HCs) were enrolled in the study.

**Results:**

CEC counts are significantly higher in both CSA and UA groups compared to the HC group [respective medians of 6.9, 10.0 and 1.5 cells/ml (*p* < 0.01)]. Further, a significant elevation of CEC count was observed in the three UA subgroups [low risk (5.3) vs. intermediate risk (10.8) vs. high risk (18.0) cells/ml, *p* < 0.001) classified in accordance to the TIMI NSTEMI/UA risk score system. From the receiver-operating characteristic curve analysis, the AUCs for distinguishing CSA and UA from HC were 0.867 and 0.938, respectively. The corresponding sensitivities were 87.5% and 84.6% and the specificities were 66.7% and 86.7%, respectively.

**Conclusions:**

Our microfluidic assay system is efficient and stable for CEC capture and enumeration. The results showed that the CEC count has the potential to be a promising clinical biomarker for the assessment of endothelial damage/dysfunction in CAD patients with angina pectoris.

## Introduction

Coronary artery disease (CAD) represents the largest proportion of cardiovascular disease and it is one of the most common causes of morbidity and mortality worldwide [1]. Currently, CAD with chronic stable angina pectoris (CSA) can be readily diagnosed with electrocardiogram (ECG), functional stress testing and coronary angiography or computed tomography (CT) [2]. On the other hand, unstable angina (UA), which may involve atherosclerosis plaque rupture or erosion [3–5] but usually with normal cardiac biomarkers and inconspicuous ECG change [6–8] is still difficult to be correctly evaluated. In addition, about 50% of individuals presented with clinical manifestations of CAD lack the traditional risk factors of elevated cholesterol, hypertension, obesity, family history, etc. [9]. Circulating endothelial cells (CECs) constitute a potential biomarker to help evaluate CAD with angina pectoris as they have been proven to assess endothelial damage/dysfunction related to the progression of atherosclerosis [5,10,11].

CECs are rare and mixed together with many complicated cellular and non-cellular components of the human blood [12,13]. The two current methods of CEC quantification include the use of immunomagnetic beads (IB) and flow cytometry (FC) [13]; however, they all could not provide a clear field for an indepth scrutiny of CECs when isolated [14]. Further, the IB technique often produces non-specific bindings between magnetic beads and non-target cells [15,16] and the FC approach suffers from low sensitivity and high variability due to its inability to detect minute amount of cells [17–19]. To address these deficiencies, innovative microfluidic contraptions have been developed for an efficient detection and reliable capture of rare cells in human blood [19,20]. Two such devices are the microfluidic magnetic disk (a combined immunomagnetic beads separation approach with an in-situ observation using a microfluidic disk) [21] and the microfluidic membrane filter (a polymer separation membrane with uniform perforations of typically 8 μm size) [22]. However, their design still had limitations of antibody-dependent capture and indiscriminate and accumulative trapping of a wide range of cells. In the membrane approach, the accumulation of cellular and non-cellular debris quickly contaminates the entire capture region making the identification of CECs challenging and also, as the membrane clogs-up, the flowrate is reduced causing the process to be time-consuming.

We adopted a fundamentally different microfluidic approach—it involves the use of a spatially staggered micropillars to spread the trapped cells to all parts of the capture chamber and prevents buildup of flow debris. The result is a clean and uncluttered field of captured cells and this clear view facilitates a real-time monitoring of the flow conditions and permits on-chip immunofluorescent staining processes for a more accurate identification outcome. Additionally, the unobstructed capture chamber allows the flowrates to be continuously maintained throughout the label-free assaying process, which contrasts greatly with the frequent flow blockages in the highly congested debris field of the membrane system. We have tested our system on 70 subjects, including healthy controls and CAD patients with CSA and UA phenotypes.

## Materials and methods

### Endothelial cell line culture and size measurements

**Fig 1:**
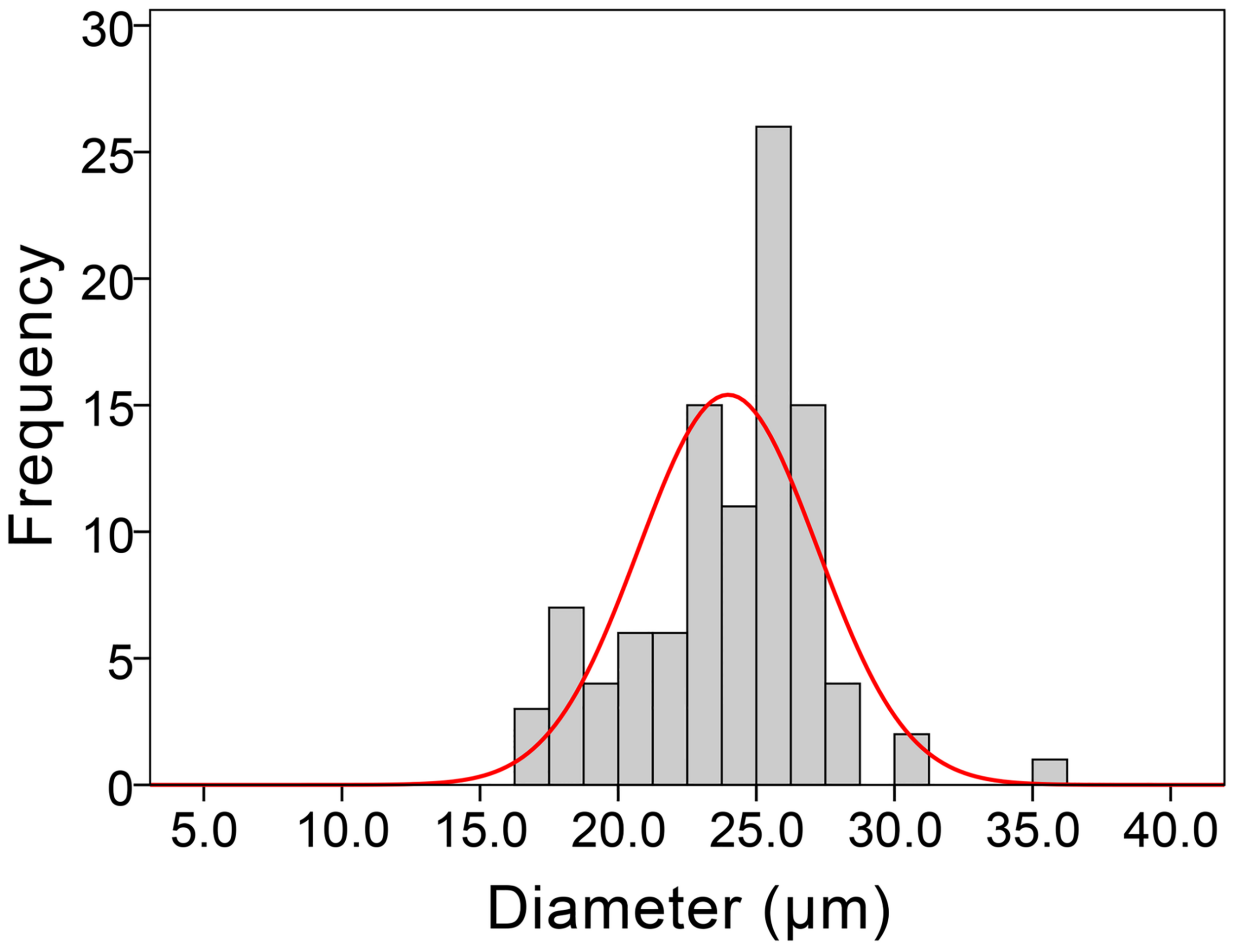
Size distribution of HUVECs. Min: 16.8μm, max: 35.3μm, mean: 24.0μm, SD: 3.2μm, skewness: -0.072 and kurtosis: 0.882.

Human umbilical vein endothelial cells (HUVECs) (purchased from American Type Culture Collection: ATCC) were checked to ensure that they were free from mycoplasma contamination and then, propagated in F12K medium (ATCC, Manassas, USA) supplemented with 10% fetal bovine serum (FBS), 1% penicillin G/streptomycin/amphotericin B, 0.1 mg/ml heparin and 0.03–0.05 mg/ml endothelial cell growth supplement, in accordance to manufacturers’ instructions. For size measurement, HUVECs were serially diluted to an expected concentration of approximately 100 cells per milliliter of phosphate buffered saline (PBS) in a 96-well plate. A sample of approximately 100 cells (enumerated by a hemocytometer) was randomly drawn from the HUVEC suspension and the diameter of each randomly selected cell was measured under microscope by using a scale ruler (Fig 1) to provide a guide on the sizing of the capture chamber design in the microfluidic chip.

### Microfluidic chip fabrication and apparatus

The microstructures of the chip were designed using AutoCAD (www.autodesk.com) and fabricated via a soft lithography approach [23]. A photo mask was first produced on a glass substrate with a 2-μm critical dimension (CapitalBio Corp., Beijing, China) and then, an 8-inch silicon wafer was spin-coated with a 25-μm thick SU-8 photoresist (MicroChem Corp., Newton, MA, USA). The depth of the micro channels was 25μm. Next, polydimethylsiloxane (PDMS) (Dow Corning, Ellsworth Adhesives, USA) was mixed with its cross-linker with a ratio of 9:1, degassed in a desiccator and cured in an oven preset at 60°C for at least 4 h. The chip assembly was completed by peeling the PDMS off the mold, punching fluidic ports and binding the PDMS to a glass slide after an oxygen plasma cleaning. Prior to the system assembly, all parts including the microfluidic chip, syringes, fluidic connectors and tubes were thoroughly washed with PBS. The assembled system was connected to a syringe pump (Longer Pump, Baoding, Hebei, China) to accurately control the preset flow rates.

### Patient characteristics and ethics

Three age-matched cohorts [39 patients with unstable angina (UA), 16 patients with chronic stable angina (CSA) and 15 healthy controls (HCs)] sourced from Peking University Hospital were enrolled in the study between April 2016 and January 2017. Patients with one or more of these conditions were excluded: pregnancy, liver disease, dialysis, malignancy, peripheral vascular disease, recent (<3 months) major trauma, active infections and/or a history of inflammatory. We considered only the CAD with angina pectoris as defined by the American College of Cardiology Foundation/ American Heart Association Guidelines and European Society of Cardiology Guidelines [2,24,25]. The diagnosis of UA included the presence of typical at-rest angina associated with acute and transient ST-segment or T-wave changes but with normal cardiac biomarkers [7,8]. Patients with cardiac chest pain, normal echocardiogram, normal cardiac biomarkers and treatment history of CAD were classified as having CSA [2]. The 39 UA patients were clinically classified into three groups (low risk group, intermediate risk group and high risk group), in accordance to the TIMI risk score for unstable angina/non-ST elevation MI. The TIMI NSTEMI/UA risk score system (Table 1) contains seven variables and the results correspond to a risk stratification (score 0–2 for low risk, score 3–4 for intermediate risk and score 5–7 for high risk) [26].

**Table 1:** Calculation of risk score using the TIMI NSTEMI/UA system.

Risk Scoring System	Inclusion Criteria	Score
**TIMI NSTEMI /UA (0–7)**	Age ≥ 65 years	1
	≥ 3 risk factors (hypertension, diabetes mellitus, family history, lipids, smoking)	1
	Known CAD (stenosis ≥ 50%)	1
	Aspirin use in past 7 days	1
	Severe angina (≥ 2 episodes within 24 hours)	1
	ST-segment deviation ≥ 0.5 mm	1
	Elevated cardiac markers	1

TIMI, Thrombolysis in Myocardial Infarction; NSTEMI, non-ST segment elevated myocardial infarction; UA, unstable angina; CAD, coronary artery disease.

This study was carried out in accordance to the Declaration of Helsinki with the protocol approved by Peking University Institutional Review Board (IRB00001052-16014). A written informed consent from all subjects was obtained prior to enrolling in the study. The detailed clinical history and diagnostic data were obtained from interviews with participants accompanied by hospital-based doctors in accordance and the permission to present the information has been received from all the participants in the study.

### Specimen collection and processing

From all enrolled patients and healthy controls, a 4-ml peripheral blood sample was drawn into an EDTA-coated anticoagulant tube. To avoid contaminating the sample with endothelial cells from the venipuncture, the first 2 ml was discarded. The peripheral blood of the UA and CSA patients were drawn within 12h after the onset of an angina attack. The samples were kept at 4°C in a biological sample box and shipped to a central lab within 1h. For UA and CSA patients, initial cardiac biomarkers [cardiac troponin I (cTnI), aspartate aminotransferase (AST), lactate dehydrogenase (LDH), creatine kinase (CK), MB isoenzyme of creatine kinase (CK-MB), α-hydroxybutyric dehydrogenase (α-HBDH)] together with either a coronary angiogram or computed tomography (CT) evidence of CAD was mandated. Before sample processing, whole blood samples were stored at 4°C on a rocking platform to prevent cell aggregation. For separation process using diluted blood, blood specimens were centrifuged at 1500 × g for 15 min, the supernatant serum discarded and the pellet re-suspended in PBS with a dilution ratio of 1:2. The resuspension sample was then pumped through the microfluidic chip using the syringe pump with the flow rate controlled at 1 ml/h. A more detailed protocol is attached as S4 File in the ‘Supporting information’ section.

### Assessment of capture efficiency and intra-assay variability

**Fig 2:**
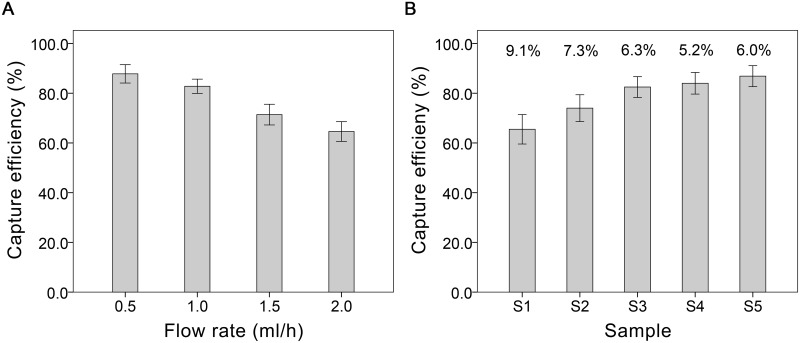
Parameters characterization of the microfluidic chip. The CE values were presented as mean (SD) of five repeat tests and the intra-assay variability was presented as the CV value of eight duplicates. (**A**) Capture efficiency test. The CE values were 87.8% (3.7%), 82.8% (2.9%), 71.4% (4.2%) and 64.6% (4.0%) at a concentration of 100 HUVECs/ml corresponding to each flow rate. (**B**) Intra-assay variability analysis. In five HUVEC-spiked samples (S1-5), the concentrations of spiked-HUVECs were 10±3, 25±8, 50±14, 100±23 and 200±34 cells/ml, respectively. The CV values were 9.1% (S1), 7.3% (S2), 6.3% (S3), 5.2% (S4) and 6.0% (S5), respectively, tested at a flow rate of 1ml/h.

To calculate the capture efficiency of the microfluidic chip, cell line HUVECs were added into whole blood samples donated by healthy donors at a concentration of approximately 100 cells per milliliter. The HUVEC-spiked samples were further processed according to the procedures (see ‘Specimen collection and processing’ section) and analyzed at four different flowrate conditions of 0.5, 1.0, 1.5 and 2.0 ml/h. Isolated cells in the microfluidic chip were first stained with fluorophore-conjugated antibodies according to the procedures (see ‘Immunofluorescence staining’ section) and enumerated under an inverted fluorescence microscope (Leica Microsystems, DM IL LED). For each flowrate, five repeated tests were performed and the capture efficiency (CE) calculated and presented as CE = [(captured HUVECs) / (HUVECs spiked in total)] × 100% (Fig 2A). To evaluate the technical variability of the microfluidic analysis, an intra-assay variability test was performed using five spiked blood samples (S1-5). Briefly, an 8 ml peripheral blood sample was drawn from each healthy donor and divided into eight duplicates. To simulate the different CEC counts in blood samples taken from different patients, replicates from the five original samples were added with different amounts of spiking (approximately 10, 25, 50, 100 and 200 HUVECs per replicate for S1-S5, respectively) and subsequently, analyzed using our microfluidic system. Intra-assay variability was presented as the CV of the capture efficiencies for each of the samples’ eight duplicates and computed via CV = (standard deviation/mean) × 100% (Fig 2B).

### Immunofluorescence staining

The captured cells in the microfluidic chip were fixed with 4% paraformaldehyde (PFA) for 30 min and washed with PBS for 15 min. They were subsequently permeabilized with 0.1% Triton X-100 for 15 min and then blocked with BlockAid Blocking Solution (Life Technologies, Eugene, Oregon, USA) for 1h to block non-specific bindings. The blocked cells were then stained with Alexa Fluor 488 conjugated anti-CD146 (Abcam, ab196448) and phycoerythrin conjugated anti-VEGFR1 (vascular endothelial growth factor receptor 1, Abcam, ab208739) for 1h. Alexa Fluor 647 conjugated anti-CD45 (Biolegend, Catalog # 304018) was used to identify leukocytes and 4’,6-diamidino-2-phenylindole (DAPI) for nuclei visualization. The antibodies used above were diluted in accordance to the manufacturers’ instructions and injected into the microfluidic chip at a flow rate of 500μL/h.

### Identification and enumeration of CECs and HUVECs via fluorescence microscopy

An inverted fluorescence microscope (Leica Microsystems, DM IL LED) with a digital microscope camera (Leica DFC450) was used to image the captured cells on the microfluidic chip. In the HUVEC-spiked blood tests, isolated HUVECs (DAPI^+^/CD146^+^/VEGFR1^+^/CD45^-^) and residuary leukocytes (DAPI^+^/CD45^+^) were distinguished. In patients’ blood tests, the cells that met the preset criteria (fluorescent expression (DAPI^+^/CD146^+^/VEGFR1^+^/CD45^-^), fluorescence intensity, cell size (15–50μm) and a well-preserved cell morphology with a distinct nucleus in a well-delimited cytoplasm) were identified as putative CECs [17,27]. Finally, the CEC and HUVEC counts were expressed as the number of the cells per milliliter of blood.

### Statistical analysis

Data were analyzed by using the Statistical Package for the Social Sciences (SPSS) version 18.0 (SPSS, Inc., Chicago, IL, USA). A two-tailed *p*-value of < 0.05 was considered to be statistically significant and < 0.01 statistically very significant for all comparisons. Applying the Kolmogorov–Smirnov test to examine the normality of all continuous data sets, appropriate parametric and non-parametric analyses were performed in the subsequent data analysis. The data were presented as mean (SD, standard deviation) when normally distributed or as median (IQR, inter-quartile range) when not normally distributed. The Kruskal–Wallis test was used for the comparison among the three data groups. For in-between group comparisons of continuous data, a two-tailed *t* test and the Mann-Whitney test were used for parametric and non-parametric data, respectively. As the data of the six cardiac biomarkers were not normally distributed, the correlation between each biomarker and the CEC count was assessed using the Spearman rank correlation. The ROC curves were then drawn according to the comparisons among HC, CSA and UA groups. The area under the curve (AUC) was computed, and the optimum ‘‘cut-off” values of CEC counts for distinguishing CSA and UA cases from the HC cases were obtained by comparing the sum value of the sensitivity and specificity.

## Results

### Microfluidic chip design for the CEC isolation

**Fig 3:**
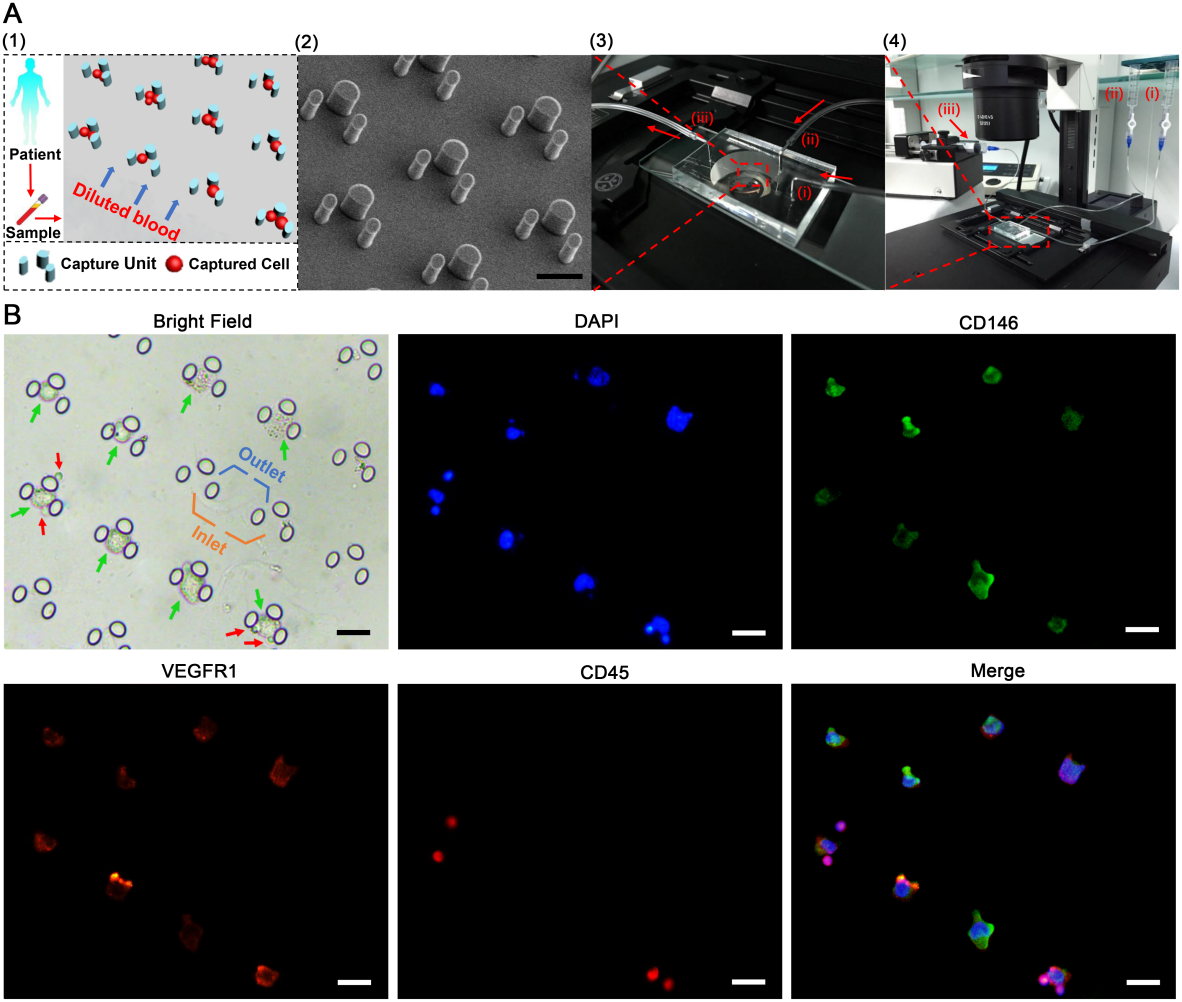
(**A**) Specially designed microfluidic system for CECs isolation: (1) the schematic diagram of blood sample processing; (2) SEM photograph of staggered capture units; (3) Microfluidic chip [(i) sample inlet, (ii) buffer inlet, (iii) waste outlet]; (4) Setup of microfluidic system [(i) blood sample reservoir, (ii) buffer reservoir, (iii) waste reservoir driven by a syringe pump]. (**B**) Immunofluorescent labeling of isolated HUVECs: HUEVECs (DAPI^+^/CD146^+^/VEGFR1^+^/CD45^-^) are marked by green arrows, residuary leukocytes (DAPI^+^/CD45^+^) are marked by red arrows; Scale bar, 50μm.

Fig 3A shows the schematic design of the chip and the integrated microfluidic system for the CEC capture. Multiple arrays of the triangular capture unit are set in the micro channels to isolate CECs while permitting other blood components to sieve through (Fig 3A (1)). The three elliptic-cylindrical micro-pillars constitute a capture unit: two for the side walls and one as a holder for the containment. The height of each pillar is 25μm, the sizes of the inlet and outlet are 30μm and 12μm, respectively (Fig 3B), which are designed based on the measured size distribution range of HUVECs (16.8–35.3μm, see Fig 1). Each chip, which is 33×22×4 mm in dimension (about the size of a Chinese dollar coin—see S6 File) contains almost 6000 staggered capture units and these units work as size-based traps to selectively immobilize the target cells as the blood flowing through.

### Chip characterization

To characterize the chip for its CE and CV values, we employed HUVECs, which are similar in size and affinity [17,28] to the CECs. To assess the CE value, we tested HUVEC-spiked samples (each sample is set at a concentration of approximately 100 HUVECs per ml) for four different flowrates of 0.5, 1.0, 1.5, 2.0 ml/h, respectively. For each flow rate, five repeated tests were performed. The captured HUVECs and residuary leukocytes were identified by immunofluorescent staining and enumerated under a microscope (Fig 3). According to the result of cell capture, the calculated CE values at the four flow rates were 87.8% (3.7%), 82.8% (2.9%), 71.4% (4.2%) and 64.6% (4.0%), respectively (Fig 2A). To balance the processing time and the CE value, we employed a flowrate of 1.0 mL/h as a comparatively ideal speed for all subsequent blood samples processing. Additionally, a detailed computer simulation of the flow environment inside the chip at the operating flowrate of 1 mL/h using the COMSOL Multiphysics software (www.comsol.com) was performed. The results obtained showed that the flow velocity ranges from 0–18 mm/s with an average shear stress at the wall of 5.1 Pa, which is comparable to the shear stress in the walls of a human artery [29]. This suggests that captured CECs were likely to maintain its integrity inside the microfluidic chip (S6 File). An intra-assay variability analysis was performed to evaluate the stability of the microfluidic analysis system. The calculated CV value of five HUVEC-spiked samples were 9.1% (S1), 7.3% (S2), 6.3% (S3), 5.2% (S4) and 6.0% (S5), respectively (Fig 2B). In the five sample tests, the CV values were all found to be < 10%, which suggest that the variability of our microfluidic analysis was limited and the specially designed microfluidic system stable.

### Baseline characteristics for subject groups and CEC count with increasing risk score

**Fig 4:**
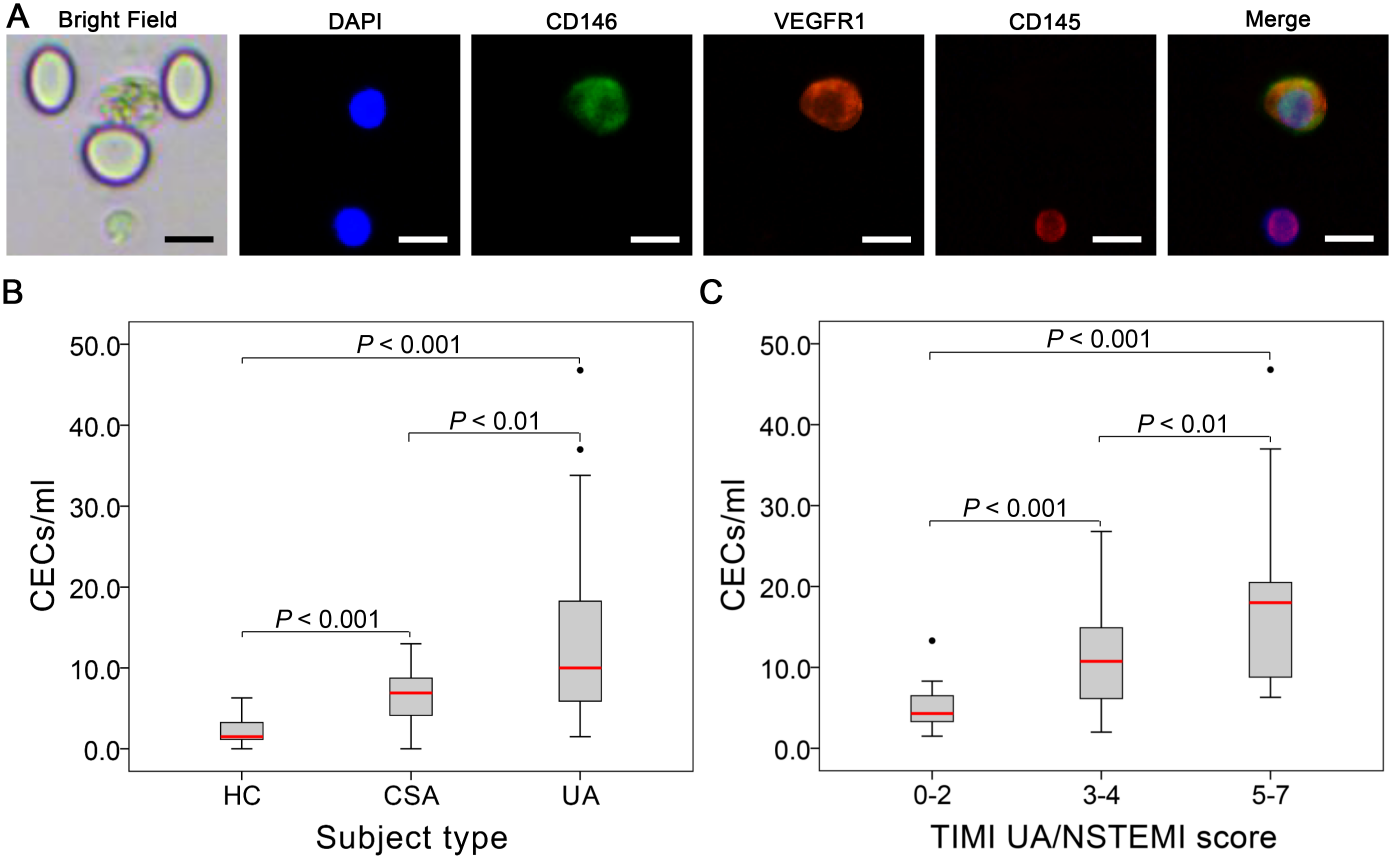
(**A**) Immunofluorescence staining of isolated CECs. Captured CECs (DAPI^+^/CD146^+^/VEGFR1^+^/CD45^-^) can be clearly observed at the single cell level, together with residuary leukocytes (DAPI^+^/CD45^+^). (**B**) Comparative CECs counts among HC group, CSA group and UA group. Data are expressed as the median with IQRs. (**C**) Comparative CEC counts with increasing TIMI UA/NSTEMI risk score. Data are expressed as the median with IQRs. P-value referred to the results of Mann-Whitney test between groups. Scale bar, 20μm.

The captured CEC (DAPI^+^/CD146^+^/VEGFR1^+^/CD45^-^) (Fig 4A) count was assessed for all the subjects (15 HCs, 16 CSA patients and 39 UA patients). The baseline characteristics of these 3 groups are listed in Table 2. As depicted, the CEC counts were significantly higher in the 2 patient groups compared with the HC group: HC [**1.5** (1.0–3.5)], CSA [**6.9** (3.8–8.9)] and UA [**10.0** (5.5–18.5)] (*p* < 0.01) (Table 2, Fig 4B). Interestingly, no significant variation in the cardiac biomarkers between CSA and UA groups was observed (Table 2).

**Table 2:** Baseline characteristics and CEC count for the three comparison groups.

Subject Factor	HC	CSA	UA	*p*-value
**N (%)**	15 (21.4)	16 (22.9)	39 (55.7)	—
**Age**	60.8 (7.0)	63.3 (8.1)	65.2 (9.1)	0.353
**Body mass index**	25.9 (3.7)	27.3 (4.1)	26.7 (4.6)	0.513
**Heart rate/minute**	72.8 (10.3)	75.6 (13.8)	81.1 (19.3)	0.312
**Males, *n* (%)**	8 (53.3)	9 (56.3)	21 (53.8)	0.869
**Risk factors, *n* (%)**				
Dyslipidemia	—	5 (31.3)	12 (30.8)	0.972
Hypertension	—	11 (68.8)	30 (76.9)	0.531
Diabetes mellitus	—	6 (37.5)	17 (43.6)	0.680
Smoking	5 (33.3)	5 (31.3)	13 (33.3)	0.954
Stroke/TIA	—	2 (12.5)	6 (15.4)	0.785
Family history of CAD	2 (13.3)	4 (25.0)	10 (25.6)	0.616
Coronary artery bypass	—	2 (12.5)	6 (15.4)	0.785
Coronary artery stents	—	4 (25.0)	11 (28.2)	0.810
**Cardiac biomarkers**				
cTnI (ng/ml)	—	<0.03	<0.06	0.056
AST (IU/L)	—	20.0 (6.1)	23.4 (10.9)	0.152
LDH (IU/L)	—	185.2 (44.1)	194.8 (39.0)	0.429
CK (IU/L)	—	116.2 (49.5)	127.3 (50.6)	0.459
CK-MB (IU/L)	—	16.7 (7.2)	20.7 (9.4)	0.132
α-HBDH (IU/L)	—	157.3 (26.9)	175.2 (33.4)	0.063
**Medication, *n* (%)**				
Aspirin	2 (13.3)	7 (43.75)	26 (66.7)	0.002
Clopidogrel	—	4 (25.0)	12 (30.8)	0.810
Beta-blocker	—	4 (25.0)	17 (43.6)	0.401
Calcium-channel blocker	—	5 (33.3)	14 (35.9)	0.744
Oral nitrate	—	5 (33.3)	12 (30.8)	0.972
ACE-inhibitor/ARB	—	6 (37.5)	18 (46.2)	0.452
Statin	2 (13.3)	12 (75.0)	31 (79.5)	0.272
**CEC/mL**	**1.5** (1.0–3.5)	**6.9** (3.8–8.9)	**10.0** (5.5–18.5)	<0.01

HC, healthy control; CSA, chronic stable angina; UA, unstable angina; TIA, transient ischemic attack; CAD, coronary artery disease; cTnI, Cardiac troponin I; AST, aspartate aminotransferase; LDH, lactate dehydrogenase; CK, creatine kinase; CK-MB, the MB isoenzyme of creatine kinase; α-HBDH, α-hydroxybutyric dehydrogenase; ACE, angiotensin-converting enzyme; ARB, angiotensin II receptor blocker. Results are expressed as mean (SD, standard deviation), or median (IQR, inter-quartile range), or as number [n (%)].

Selecting the UA group in particular, there was a significant increase in the CEC count with an increasing TIMI NSTEMI/UA risk score for the three categories of low, intermediate and high risk. The results are as follows: low risk category [5.3 (3.1–7.0)], intermediate risk category [10.0 (5.5–18.5)] and high risk category [18.0 (8.7–22.4)] (*p* < 0.001) (Table 3, Fig 4C).

**Table 3:** Comparing CEC count with an increasing TIMI UA/NSTEMI risk score for the 3 categories.

TIMI NSTEMI/UA Risk score	Low	Intermediate	High	*p*-value
**Score**	0–2	3–4	5–7	**—**
***N* (%)**	14 (35.9)	15 (38.5)	10 (25.6)	—
**CEC/ml**	5.3 (3.1–7.0)	10.8 (5.8–15.4)	18.0 (8.7–22.4)	<0.001

TIMI, Thrombolysis in Myocardial Infarction; NSTEMI, non-ST segment elevated myocardial infarction; UA, unstable angina; CEC, circulating endothelial cells.

### Correlations between CEC count and typical cardiac biomarkers

**Fig 5:**
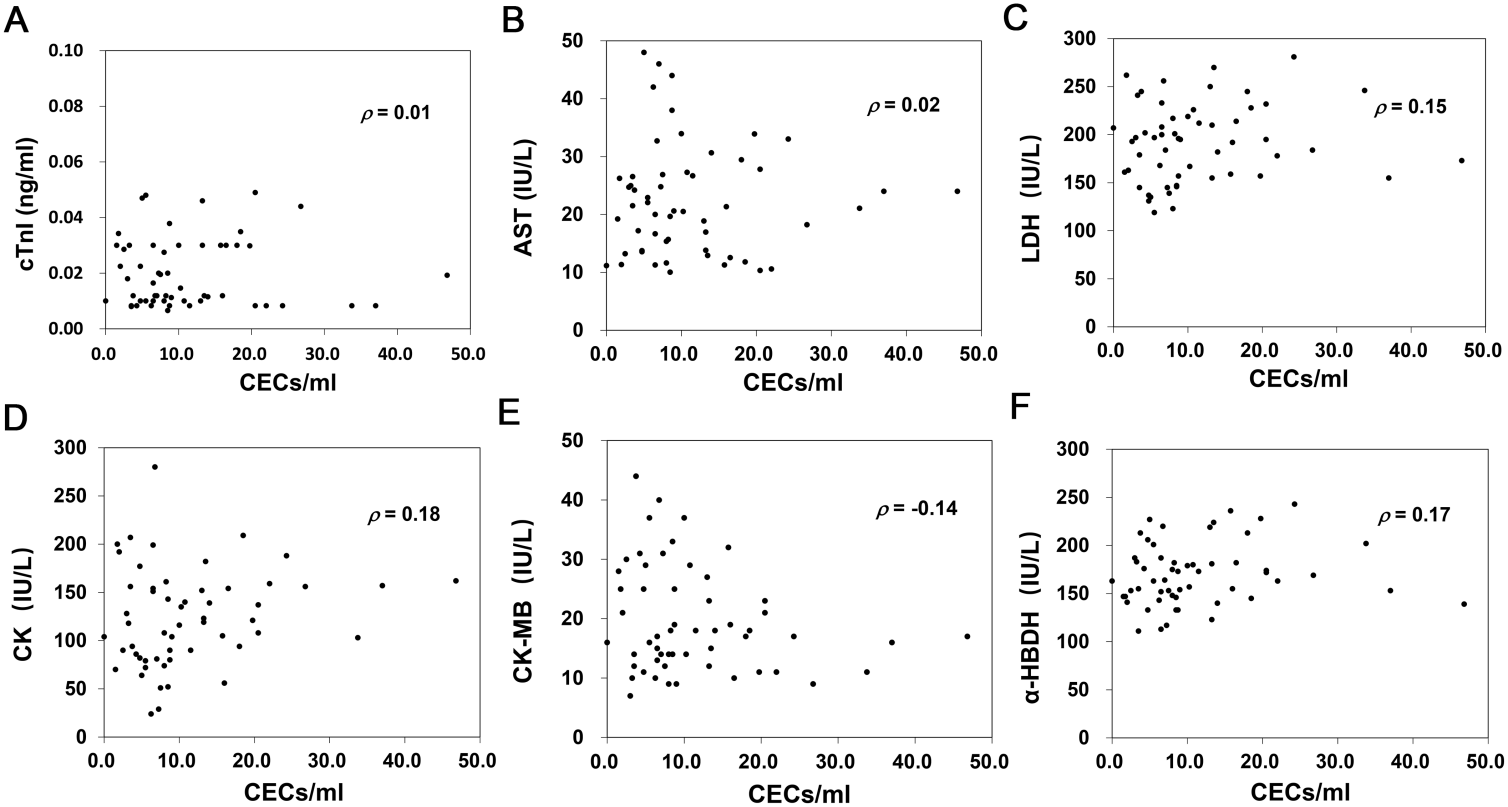
Correlations between CEC count and typical cardiac biomarkers. Spearman rank correlation coefficient (*ρ*) was used to assessed the correlation between CEC counts in CAD patients with angina pectoris and each initial presenting serum cardiac biomarkers. (**A**) No correlation was observed between CEC count and cTnI value (*ρ* = 0.01, *p* = 0.987). (**B**) No correlation was observed between CEC count and AST value (*ρ* = 0.02, *p* = 0.910). (**C**) No correlation was observed between CEC count and LDH value (*ρ* = 0.15, *p* = 0.271). (**D**) No correlation was observed between CEC count and CK value (*ρ* = 0.18, *p* = 0.181). (**E**) No correlation was observed between CEC count and CK-MB value (*ρ* = -0.14, *p* = 0.326). (**F**) No correlation was observed between CEC count and α-HBDH value (*ρ* = 0.17, *p* = 0.212).

The Spearman rank correlation coefficient (*ρ*) was used to assess the correlation between the CEC count and the 6 cardiac biomarkers. As shown in Fig 5, the result of correlation is as follows: cTnI with CEC count (*ρ* = 0.01, *p* = 0.987), AST with CEC count (*ρ* = 0.02, *p* = 0.910), LDH with CEC count (*ρ* = 0.15, *p* = 0.271), CK with CEC count (*ρ* = 0.18, *p* = 0.181), CK-MB with CEC count (*ρ* = -0.14, *p* = 0.326), and α-HBDH with CEC count (*ρ* = 0.17, *p* = 0.212). It is clear that there was no evidence of correlation between the CEC count and each of the 6 biomarkers.

### Receiver-operating characteristic curve (ROC) analyss

**Fig 6:**
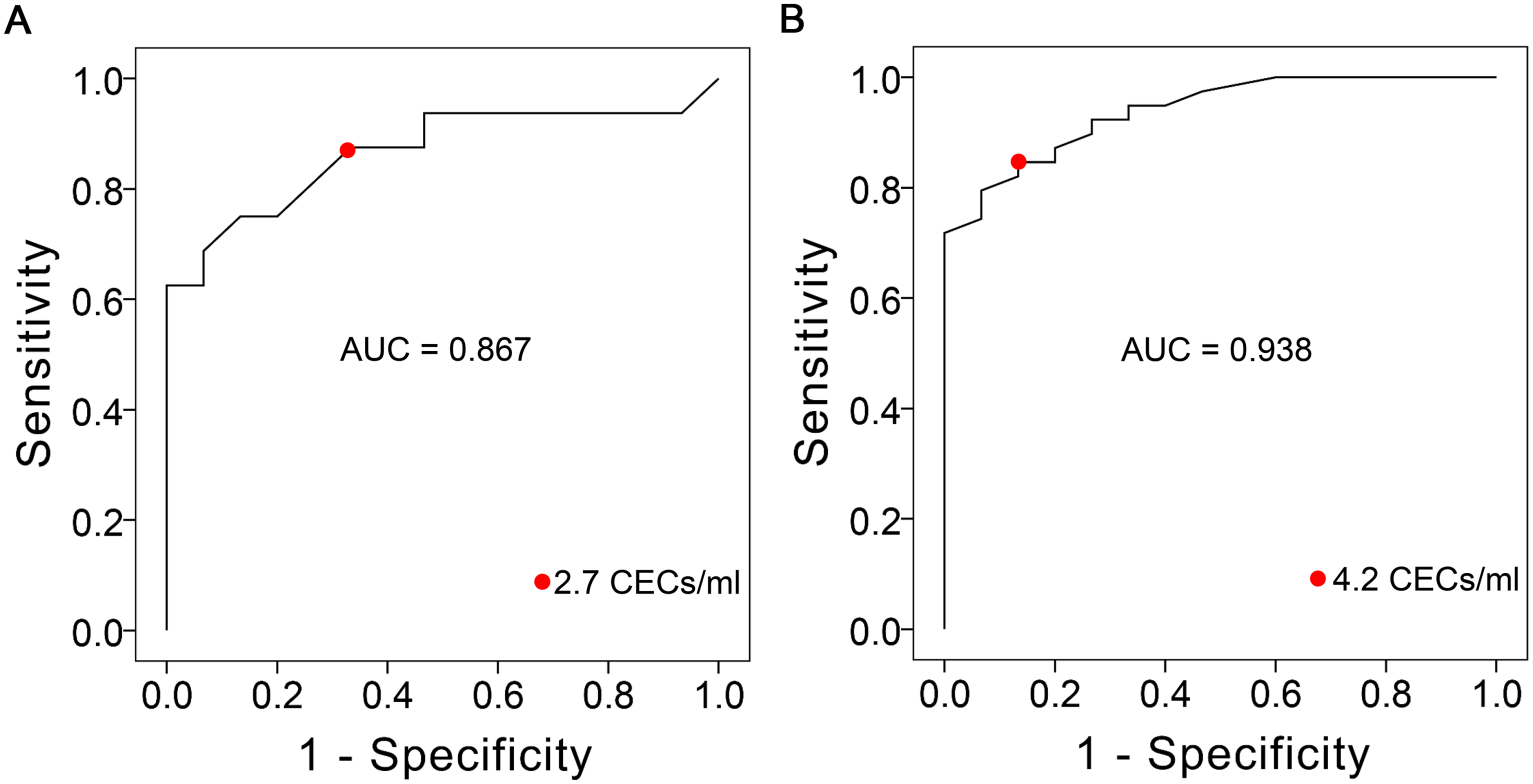
Receiver-operator characteristic curves illustrating the predictive accuracy of CEC count for the diagnosis of CAD with angina pectoris. (**A**) AUC was equal to 0.867 (*p* < 0.01). The red point represented a classification cutoff value of 2.7 CEC/ml, which is associated with a sensitivity of 87.5% and specificity of 66.7% to accurately differentiate a CSA case from a healthy control. (**B**) AUC was equal to 0.938 (*p* < 0.001). The red point represented a classification cutoff value of 4.2 CEC/ml, which is associated with a sensitivity of 84.6% and specificity of 86.7% to accurately differentiate a UA case from a healthy control.

To determine whether the CEC count can be used as a complementary biomarker for the diagnosis of CAD with angina pectoris, we performed a ROC analysis of the 3 groups: HC, CSA and UA. From the ROC curves depicted in Fig 6, the AUC (the area under the curve) for the diagnosis of CSA and UA were 0.867 (*p* < 0.01) and 0.938 (*p* < 0.001), respectively. The corresponding sensitivities were 87.5% and 84.6% and the specificities were 66.7% and 86.7%. The rational cutoff values (diagnosis threshold) were 2.7 CEC/ml and 4.2 CEC/ml, respectively.

## Discussion

CECs, which are mature cells exfoliated from the intima monolayer of a blood vessel during an event involving endothelial damage/dysfunction constitute less than a thousandth of a percent of the peripheral blood cells. It is this technical challenge of extreme rarity detection that is generating innovative quantification techniques to assist in the development of CECs as a clinically relevant biomarker for many diseases, CAD included. Our approach focused on the capture of CECs with an uncluttered field of view—this significantly increased the reliability and reproducibility of the in-situ enumeration of these rare cells. As shown in Fig 3B, spiked HUVECs were trapped in a staggered arrangement that produced a clean capture chamber for an easy and convenient on-chip fluorescent labeling. Our chip characterization results showed a high CE value 82.8% (2.9%) and a low CV value < 10%—two metrics to indicate that our microfluidic system is both efficient and stable for use as a potential liquid biopsy tool for the diagnostic and prognostic assessment of CAD with angina pectoris.

In our work, we used CD146 and VEGFR1 simultaneously for CEC identification; CD146 is a well-described adhesion marker, which is expressed in all types of human endothelial cells [12, 30–32], while VEGFR1 is a typical tyrosine kinase receptor in the extracellular region with a high specificity for vascular endothelial cells [33–36]. Hence, it makes good sense to adopt a combined CD146 and VEGFR1 immunostaining approach together with the preset criteria presented in the ‘Materials and methods’ section for an enhanced detection accuracy of CECs.

In our results, we observed a significantly higher CEC count in correlation to the severity of CAD with angina pectoris (Table 2, Fig 4B), while no significant variation was found in the conventional cardiac biomarkers (Table 2). Further, a significant increase in the CEC count with an increasing TIMI NSTEMI/UA risk score was obtained for the three subgroups of UA (Table 3, Fig 4C). No correlation was found between the CEC count and each of the six cardiac biomarkers (Fig 5). These results reinforce our contention that the CEC count has the potential to be a relatively independent marker for the clinical assessment of CAD with angina pectoris when compared with the typical cardiac biomarkers.

Additionally, we provided two different cutoff values to respectively, distinguish the CSA and UA cases from the HC cases. To accurately classify the CSA case from control, the rational cutoff value was 2.7 cells/ml (with AUC of 0.867, sensitivity of 87.5% and specificity of 66.7%), which shows that CECs in healthy controls are extremely rare (< 2.7 cells/ml). This result is comparable to the CEC count of < 3 cells/ml in healthy individuals as reported by Boos *et al*. [13]. To accurately differentiate UA case from control, the rational cutoff value was 4.2 cells/ml (with AUC of 0.938, sensitivity of 84.6% and specificity of 86.7%), which is reasonable when compared with the cutoff of 9 cells/ml (with AUC of 0.95, sensitivity of 90% and specificity of 93%) for the diagnosis of acute myocardial infarction as reported by Damani *et al*. [14] and the cutoff of 7 cells/ml (with AUC of 0.82, sensitivity of 92.4% and specificity of 44.8%) for diagnosis of acute coronary syndromes as reported by Boos *et al*. [8].

Although our work demonstrates that the efficiency and stability of our microfluidic approach for CEC detection are satisfactory, there are still some limitations that need to be further improved before the method can be used in a clinical environment. First, the flow rate and capture efficiency may be further enhanced by fabricating multiple microchannels with sophisticated design of capture chamber. Second, the immunolabeling method we proposed for the CEC identification may not be able to identify every phenotype of CECs—a more comprehensive staining method should be explored based on our label-free isolation of CECs. Third, to get a more accurate understanding of the molecular biological features of CECs in CAD with angina pectoris, a more precise analysis of single cell sequencing on CECs can be performed after using our microfluidic method to harvest CECs.

## Conclusion

In this work, we have demonstrated an efficient and stable microfluidic system for a label-free capture and on-chip immunofluorescence enumeration of CECs. The evidence to support a consistent relationship between the severity of CAD with angina pectoris and CEC count is clearly presented. In addition, no obvious evidence of correlation was found between the CEC count and the 6 well-accepted cardiac biomarkers, therefore, CEC could be potentially used as a relatively independent marker to evaluate CAD with angina pectoris and perhaps, aid in the prediction of an imminent risk of a catastrophic cardiovascular event.

## Supporting information

S1 File PLOS ONE clinical studies checklist. (PDF)

S2 File The ethics approval. (PDF)

S3 File STROBE checklist for observational studies. (PDF)

S4 File Detailed protocol for isolation and identification of circulating endothelial cells via microfluidic approach. (PDF)

S5 File Interview guide for clinical data collection. (PDF)

S6 File Supplementary chip characterization. (PDF)

S7 File Original dataset. (PDF)
