# Supplementary material for: Microfluidic assay of circulating endothelial cells in coronary artery disease patients with angina pectoris
Source: PLoS One. 2017 Jul 13;12(7):e0181249. doi: 10.1371/journal.pone.0181249 (PMC5509377; doi:10.1371/journal.pone.0181249)
Supplement: S2 File — (PDF) [file pone.0181249.s002.pdf]

Peking University Institutional Review Board

Approval Letter

No.: IRB00001052-16014

Protocol Accepting No.	2016033（工学院）		
Protocol Title	Microfluidic Assay of Circulating Endothelial Cells from Peripherial Blood of Coronary Artery Disease Patients with Angina Pectoris		
Funding Source	Research Study of Graduate Student		
Principal Investigator	Pingchou Han		
PI's Department	College of Engineering, Peking University		
review Category	Primary Review	review Method	Convened
Documents Reviewed	Refer to attachment "list of documents reviewed"		

Review Decision:

According to **WMA *Declaration of Helsinki*, CIOMS *International Ethical Guidelines for Biomedical Research Involving Human Subject*, *Chinese Good Clinical Practice*, National Health and Family Planning Commission (NHFPC) *Guidance on Ethical Review of Biomedical Research involving Human Subjects*, CFDA *Ethical Review Guidelines on Clinical Trial* and related regulatory requirements set forth by international and national laws, regulations, guidelines, as well as policies and procedures at Peking University Human Research Protection Program,** Peking University Institutional Review Board has determined that the protocol "***Microfluidic Assay of Circulating Endothelial Cells from Peripherial Blood of Coronary Artery Disease Patients with Angina Pectoris***" meets the criteria for approval.

Please conduct the study in compliance with the approved protocol and ensure the safety and welfare of the participants.

1. Submit "Application of Amendment" once there is any change of the study, including but not limited to the change of principle investigator, protocol, Informed Consent Form, Clinical Report Form, Questionnaires or recruitment materials;
2. Please report to PU IRB in time as required or planned in approved protocols once any adverse events or safety issues identified;
3. Submit "Non-compliance Report" once the investigator fails to comply with the approved protocol, jeopardize subject's safety and welfare, or the scientific validity of the study;
4. If the study is prematurely terminated or suspended for any reason, promptly submit

the "Termination & Suspension Report" to the IRB;<br>5. Submit Closure Report when study is completed;<br>6. This approval letter is valid for one year, please submit continuing review application at least 30 calendar days before the expiration date.			
Approval Date	March 29, 2016	Expire date	March 29, 2017
Chair Signature	Yali Cong	Sign Date	March 31, 2016

Attachment: List of Documents Reviewed and Approved:

No.	Item (please specify version date and version number)
1.	Initial Review Application Form
2.	Research Protocol (version 01, version date 20160317)
3.	List of Key Research Personnel
4.	PI's CV
5.	CV of Co-PI
6.	Informed Consent Form (version 01, version date 20160317)
7.	Clinical Data Collection Form
8.	GCP Certificate of PI
