# Supplementary material for: Microfluidic assay of circulating endothelial cells in coronary artery disease patients with angina pectoris
Source: PLoS One. 2017 Jul 13;12(7):e0181249. doi: 10.1371/journal.pone.0181249 (PMC5509377; doi:10.1371/journal.pone.0181249)
Supplement: S4 File — (PDF) [file pone.0181249.s004.pdf]

The Detailed Protocol for Isolation and Identification of Circulating Endothelial Cells in Human Peripheral Blood Samples

Description

Circulating endothelial cells (CECs) are widely reported as a promising biomarker of endothelial damage/dysfunction in coronary artery disease (CAD). In this protocol, we demonstrated a novel microfluidic assay which involved an in-situ enumeration and immunofluorescent identification (DAPI+/CD146+/VEGFR1+/CD45-) of CECs in human peripheral blood samples.

Materials

Regents

- Polydimethylsiloxane (PDMS) (Dow Corning, Ellsworth Adhesives, cat. no, 184, USA)
- Sterile PBS, 1× (Wisent, cat. no. 311-010-CL, Canada)
- EDTA (Xilong Chemical, China)
- Paraformaldehyde (for cell fixation; Sinopharm Chemical Reagent, cat. no. 80096692, China) ! CAUTION This chemical is a potential carcinogen and can be very hazardous in case of skin contact. Wear gloves, goggles, a lab coat and a mask when handling this chemical.
- Triton X-100 (for cell permeabilization; Sigma-Aldrich, cat. no. T9284, USA) ! CAUTION Wear gloves, goggles, a lab coat and a mask when handling this chemical.
- BlockAid Blocking Solution (for cell blocking; Life Technologies, USA)
- Alexa Fluor 488 conjugated anti-CD146 (Abcam, ab196448, UK)
- Phycoerythrin conjugated anti-VEGFR1 (Abcam, ab208739, UK)
- Alexa Fluor 647 conjugated anti-CD45 (Biolegend, Catalog # 304018, USA)

- DAPI (Life Technologies, USA)

Equipment

- Microfluidic chips (Lab manufacture)
- Inverted fluorescence microscope (Leica Microsystems, DM IL LED, German)
- PharMed BPT tubing (Cole-Parmer, cat. no. 96880-00, USA)
- Precision syringe tips (Nordson EFD, cat. no. 7018272, USA)
- Four-Dimentional Roatating Mixer (Kylin-Bell Lab Instruments, BE1100, China)
- Stopcocks with Luer connection, 1-way male lock (Cole-Parmer, cat. no. 30600-05, USA)
- Sterile disposable syringes, 5 mL and 10 mL (as recovery buffer and waste vessels; Jiangsu Zhiyu Medical Instrument, China)
- Syringe pump (Longer Pump, TS-1B/W0109-1B, UK)
- Low speed centrifuge (Beijing Era Beili Centrifuge, DT5-2, China)
- Micropipettes, 2.5 µL, 200 µL and 1000 µL (Eppendorf Research Plus, German)
- Refrigerator (Haier, China)

Experimental procedures

Here is the flow chart to describe the whole workflow to isolate and identify CECs from human peripheral blood via microfluidic method.

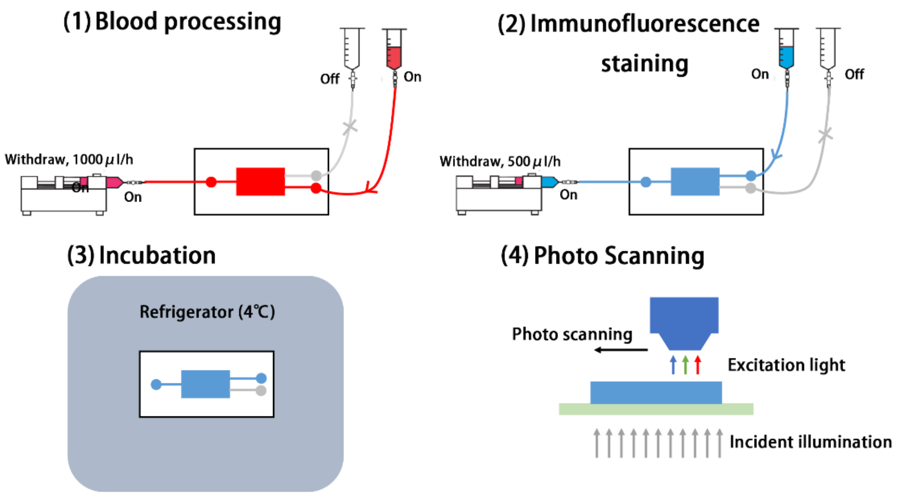

Fig S1. Flow chart of CECs isolation and identification procedures.

Blood Sample Collection and Pretreatment

1. Collect about 4 mL peripheral blood into EDTA-coated tubes from each subjects with the first 2mL discarded and ship samples to the central lab within 1h.
2. Put the samples on a rocking platform and keep them at 4℃.
3. Centrifuge the samples at 1500 × g for 15 min and discard the supernatant serum.
4. Re-suspend the rest of the blood in sterile PBS with a dilution ratio of 1:2.

Blood Sample Processing

1. Make the microfluidic system ready (According to the Fig 3A (4), connect the chips, tubes, reservoirs, stopcocks, syringes, pumps together and wash the whole circuit with sterile PBS.
2. Add the diluted blood into sample reservoir, open the sample stopcock; add 250μl PBS into the buffer reservoir and close the stopcock.
3. Set the pump at the constant withdraw flowrate of 1ml/h.

   ! CAUTION Wear gloves, goggles, a lab coat and a mask when handling blood to prevent infections. ! CAUTION Blood from patients must be obtained in compliance with the institutional and national guidelines. ▲ CRITICAL The maximum time to processing is 4 hours, above which blood coagulation may ensue.

Immunofluorescence Staining

1. After sample processing, close the sample stopcock and open the buffer stopcock, set the flowrate at 500μl/h and wash the whole circuit for 30 min with PBS.
2. Add 250μl 4% paraformaldehyde (PFA) to buffer reservoir and withdraw it at the flowrate of 500μl/h to fix the captured cells for 30 min, afterwards wash with PBS for 15 min.

3. Permeabilize the cells with 0.1% Triton X-100 (add 125μl) for 15 min and wash with PBS for 15 min.
4. Block with BlockAid Blocking Solution for 1h (add 500μl) and wash with PBS for 15 min.
5. Stain the captured cells with DAPI, Alexa Fluor 488 conjugated anti-CD146, phycoerythrin conjugated anti-VEGFR1 and Alexa Fluor 647 conjugated anti-CD45 for 1h (both dilute to 1:100 according to the manufacturers' instructions)
6. Stop the system, close all the stopcocks, take down the chip and put it into the 4℃ refrigerator for overnight incubation.

Identification and Enumeration of CECs and HUVECs

1. Load the microfluidic chip on the system and wash the capture region for 15 min to minimize the fluorescence background noise.
2. Put the chip under the invert fluorescence microscope and use different fluorescence pattern to scan all the capture region carefully.
3. Enumerate the CECs according to the preset criteria (fluorescent expression of DAPI+/CD146+/VEGFR1+/CD45-, fluorescence intensity, cell size (15-50μm) and a well-preserved cell morphology with a distinct nucleus in a well-delimited cytoplasm) and record the counting result.
