# Supplementary material for: Microfluidic assay of circulating endothelial cells in coronary artery disease patients with angina pectoris
Source: PLoS One. 2017 Jul 13;12(7):e0181249. doi: 10.1371/journal.pone.0181249 (PMC5509377; doi:10.1371/journal.pone.0181249)
Supplement: S5 File — (PDF) [file pone.0181249.s005.pdf]

Interview Guide for Clinical Data Collection

Patient ID: ____________ Gender: ☐male/☐female

Case file No. __________ Date: ____mm ____dd ____yy

History of present illness (HPI): ____

____

Past medical history (PMH):

1. Smoking history: ____
2. Hypertension history: _______________ Medication: ________________
3. Dyslipidemia history: _______________ Medication: ________________
4. Diabetes mellitus history: ____________ Medication: ________________
5. Stroke/TIA history: _________________ Medication: ________________
6. Family history of CAD: ____
7. CABG/ coronary stents : _____________ Medication: ________________

Exclusion criteria: pregnancy, liver disease, dialysis, malignancy, other severe peripheral vascular disease, recent (<3 months) major trauma, active infections and/or a history of inflammatory.

Physical index:

Heart rate: ________bpm Body mass index (BMI): ___________

ECG manifestations: ____

____

Coronary artery CT/ angiography: ____________________________________

____

Cardiac biomarkers:

cTnI: _____ng/ml AST: ______IU/L LDH: ______IU/L

CK: ______ IU/L CK-MB: ______IU/L α-HBDH: ______ IU/L

Medication in hospital:

Aspirin: ____________ Clopidogrel: ___________ Beta-blocker: ___________

Calcium-channel blocker: ___________ Oral nitrate: ___________

ACE-inhibitor/ARB: _______________ Statin: _______________
