# Supplementary material for: Microfluidic assay of circulating endothelial cells in coronary artery disease patients with angina pectoris
Source: PLoS One. 2017 Jul 13;12(7):e0181249. doi: 10.1371/journal.pone.0181249 (PMC5509377; doi:10.1371/journal.pone.0181249)
Supplement: S6 File — (PDF) [file pone.0181249.s006.pdf]

Supplementary Chip Characterization

As shown in Fig S1, our microfluidic chip is 33×22×4 mm in dimension (about the size of the Chinese dollar coin).

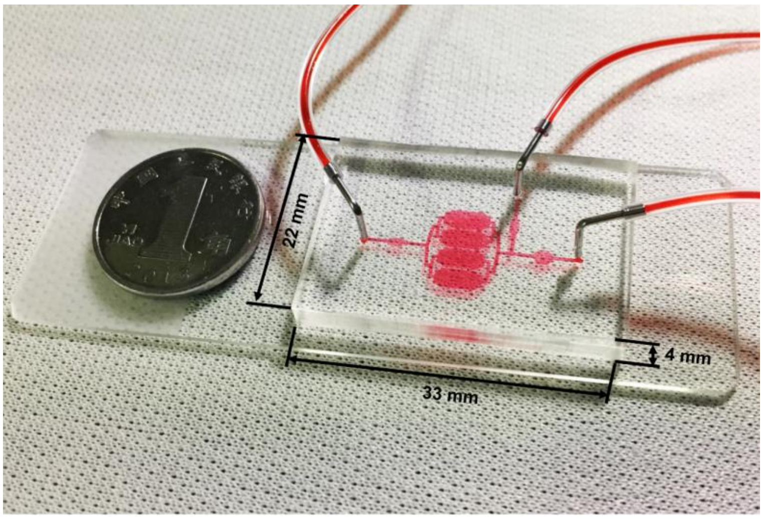

Fig S1. Microfluidic chip for blood processing.

To properly characterize the flow profile inside the chip, we carried out a detailed computer simulation to estimate the flow velocity and shear stress at the operating flowrate of 1 mL/h using the COMSOL Multiphysics (www.comsol.com). The results showed the velocity and wall shear stress range from 0-18 mm/s and 0-10 Pa, respectively (Fig S2). The lower velocity field around the capture units facilitates the entry of bigger cells, such as CECs (Fig S2 A) and the high velocity field in the gaps of adjacent capture units facilitates the transit of smaller cells (RBCs) and debris along the streamlines (Fig S2 A).

Fig S2 B depicts the wall shear stress profile surrounding the capture units, which has direct influences on the viability, cell function and integrity of endothelial cells [1]. When contrasting to the shear stress state in human artery (1.0-7.0 Pa) [2], the estimated average wall shear stress (5.1 Pa) around the capture units was comparable. This indicated that CECs were likely to maintain its integrity while they were captured in the microfluidic chip.

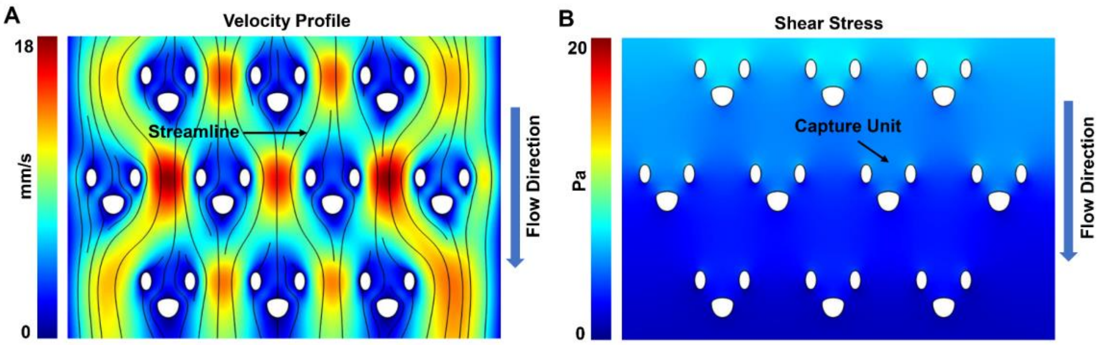

Fig S2. Computational analysis of the flow and shear stress around the capture units at the operating flowrate of 1 mL/h. (**A**) Velocity profile when isolating cells. (**B**) Shear stress profile when isolating cells.

1. Oren Traub, Bradford C. Berk. Laminar shear stress: mechanisms by which endothelial cells transduce an atheroprotective force. Arteriosclerosis Thrombosis & Vascular Biology. 1998; 18(5):677-685. doi: 10.1161/01.ATV.18.5.677 PMID: 9598824

2. Malek AM, Alper SL, Izumo S. Hemodynamic Shear Stress and Its Role in Atherosclerosis. JAMA. 1999; 282(21): 2035-2042. doi: 10.1001/jama.282.21. 2035 PMID: 10591386
