# Supplementary material for: Microfluidic assay of circulating endothelial cells in coronary artery disease patients with angina pectoris
Source: PLoS One. 2017 Jul 13;12(7):e0181249. doi: 10.1371/journal.pone.0181249 (PMC5509377; doi:10.1371/journal.pone.0181249)
Supplement: S7 File — (PDF) [file pone.0181249.s007.pdf]

Original dataset

Table S1. Size distribution of HUVECs.

No.	Diameter/μm	No.	Diameter/μm	No.	Diameter/μm	No.	Diameter/μm
1	25.4	26	20.1	51	26.3	76	23.5
2	24.5	27	31.1	52	21.5	77	25.2
3	35.3	28	19.7	53	24.6	78	25.1
4	22.8	29	26.5	54	26.6	79	25.4
5	23.2	30	26.8	55	25.4	80	25.3
6	19.2	31	30.9	56	25.4	81	23.2
7	18.2	32	26.8	57	25.4	82	24.9
8	26.2	33	27.2	58	26.6	83	22.8
9	27.7	34	26.5	59	25.7	84	17.2
10	16.8	35	26.5	60	21.4	85	23.5
11	19.1	36	25.1	61	25.3	86	24.1
12	19.7	37	20.1	62	21.1	87	23.9
13	18.2	38	25.2	63	22.8	88	24.9
14	26.9	39	20.6	64	22.1	89	23.8
15	17.9	40	17.2	65	18.3	90	23.4
16	27.4	41	25.5	66	25.8	91	23.2
17	26.2	42	26.3	67	25.2	92	24.6
18	26.9	43	20.5	68	25.1	93	23.7
19	18.2	44	24.6	69	21.9	94	23.7
20	28.2	45	22.9	70	25.7	95	20.1
21	26.6	46	27.9	71	25.5	96	25.1
22	27.9	47	25.7	72	25.3	97	25.2
23	26.6	48	21.4	73	23.2	98	23.8
24	22.1	49	18.6	74	25.7	99	23.4
25	23.2	50	26.0	75	24.5	100	18.6

Table S2. Capture efficiency test.

Sample		Flow rate (ml/h)	Spiked HUVECs/ml	Captured HUVECs/ml	DE (%)	Median (SD, %)
P1	P1-1	0.5	112	97	86.6	87.8 (3.7)
	P1-2		107	91	85.0	
	P1-3		91	85	93.4	
	P1-4		87	78	89.7	
	P1-5		121	102	84.3	
P2	P2-1	1.0	88	75	85.3	82.8 (2.9)
	P2-2		103	81	78.6	
	P2-3		114	98	86.0	
	P2-4		94	78	83.0	
	P2-5		117	95	81.2	
P3	P3-1	1.5	91	65	71.5	71.4 (4.2)
	P3-2		97	64	66.0	
	P3-3		123	92	74.8	
	P3-4		83	57	68.7	
	P3-5		108	82	75.9	
P4	P4-1	2.0	119	76	63.9	64.6 (4.0)
	P4-2		93	65	69.9	
	P4-3		84	52	61.9	
	P4-4		87	52	59.8	
	P4-5		105	71	67.6	

Table S3. Intra-assay variability analysis.

Sample		Spiked HUVECs (ml)	Captured HUVECs (ml)	DE (%)	Mean (%)	SD (%)	CV (%)
S1	S1-1	11	7	63.6	65.5	5.9	9.1
	S1-2	14	8	57.1			
	S1-3	8	6	75.0			
	S1-4	9	5	55.6			
	S1-5	13	9	69.2			
	S1-6	12	7	58.3			
	S1-7	13	9	69.2			
	S1-8	10	6	60.0			
S2	S2-1	27	20	74.1	74.0	5.4	7.3
	S2-2	32	21	65.6			
	S2-3	21	15	71.4			
	S2-4	19	15	78.9			
	S2-5	23	19	82.6			
	S2-6	26	20	76.9			
	S2-7	30	21	70.0			
	S2-8	18	13	72.2			
S3	S3-1	41	33	80.5	82.5	5.2	6.3
	S3-2	47	41	87.2			
	S3-3	58	48	82.8			
	S3-4	55	42	76.4			
	S3-5	64	56	87.5			
	S3-6	36	30	83.3			
	S3-7	61	48	78.7			
	S3-8	52	44	84.6			
S4	S4-1	112	94	83.9	84.0	4.3	5.2
	S4-2	123	113	91.9			
	S4-3	108	86	79.6			
	S4-4	77	61	79.2			
	S4-5	83	67	80.7			
	S4-6	89	78	87.6			
	S4-7	94	80	85.1			
	S4-8	86	71	82.6			
S5	S5-1	234	201	85.9	86.9	5.2	6.0
	S5-2	217	206	94.9			
	S5-3	166	144	86.7			
	S5-4	187	157	84.0			
	S5-5	175	144	82.3			
	S5-6	191	168	88.0			
	S5-7	204	184	90.2			
	S5-8	225	187	83.1			

Table S4. Cardiac biomarkers and CEC count for the three comparison groups.

Group	NO.	Gender	Age	CECs/ml	cTnI (ng/ml)	AST (IU/L)	LDH (IU/L)	CK (IU/L)	CK-MB (IU/L)	α-HBDH (IU/L)
HC	001	M	63	1.3	-	-	-	-	-	-
	002	M	68	4.0	-	-	-	-	-	-
	003	F	52	3.8	-	-	-	-	-	-
	004	M	59	1.5	-	-	-	-	-	-
	005	F	67	2.0	-	-	-	-	-	-
	006	F	58	0.5	-	-	-	-	-	-
	007	F	53	2.3	-	-	-	-	-	-
	008	M	69	3.0	-	-	-	-	-	-
	009	M	72	1.5	-	-	-	-	-	-
	010	M	61	2.3	-	-	-	-	-	-
	011	M	66	6.3	-	-	-	-	-	-
	012	F	58	1.0	-	-	-	-	-	-
	013	M	64	0.5	-	-	-	-	-	-
	014	F	50	4.8	-	-	-	-	-	-
	015	F	52	3.5	-	-	-	-	-	-
	016	M	63	8.0	0.01	15	123	74	14	148
	017	F	61	10.3	0.01	20	167	135	14	157

CSA	018	F	63	3.5	0.01	27	145	156	14	111
	019	F	54	2.3	0.01	11	207	104	16	163
	020	F	65	6.5	0.01	11	233	154	13	187
	021	M	53	4.8	0.01	14	131	82	11	133
	022	F	68	9.0	0.01	21	195	104	9	154
	023	F	56	10.8	0.01	27	226	140	29	180
	024	F	69	6.5	0.02	20	200	199	15	152
	025	M	69	3.0	0.02	25	197	128	7	187
	026	F	59	5.5	0.01	22	197	79	16	163
	027	M	49	7.5	0.02	27	139	51	12	153
	028	M	74	1.8	0.03	26	262	200	25	147
	029	M	80	8.5	0.02	10	146	52	14	146
	030	F	67	13.0	0.01	19	250	152	27	219
	031	M	62	7.3	0.02	25	145	49	31	117
	032	F	53	5.5	0.05	23	119	72	37	201
	033	F	66	11.5	0.01	27	212	90	18	173
	034	M	78	15.8	0.03	11	159	105	32	236
	035	F	61	10.0	0.03	34	219	116	37	179

UA	036	M	62	13.3	0.03	14	210	119	12	181
	037	F	71	8.8	0.04	44	196	80	19	173
	038	M	51	22.0	0.01	11	178	159	11	163
	039	F	76	26.8	0.04	18	184	156	9	169
	040	M	72	18.5	0.03	12	228	209	18	145
	041	M	71	19.8	0.03	34	157	121	11	228
	042	F	57	2.5	0.03	13	193	90	30	153
	043	F	62	6.8	0.02	24	173	162	17	139
	044	M	57	8.0	0.03	12	217	108	9	175
	045	F	67	20.5	0.05	10	232	108	21	172
	046	F	62	3.3	0.03	25	241	118	10	183
	047	F	60	16.0	0.01	21	192	56	19	155
	048	F	76	13.3	0.05	17	155	123	23	123
	049	F	53	16.5	0.03	13	214	154	10	182
	050	F	56	6.5	0.03	17	208	151	17	113
	051	F	62	8.8	0.01	38	157	90	25	133
	052	F	55	14.0	0.01	31	182	139	18	140
	053	M	58	18.0	0.03	29	245	94	17	213

054	F	60	33.8	0.01	21	246	103	11	202
055	F	80	20.5	0.01	28	195	137	23	174
056	F	79	37.0	0.01	24	155	157	16	153
057	F	62	8.3	0.01	16	201	161	18	182
058	F	59	1.5	0.03	19	161	70	28	147
059	F	72	3.8	0.01	24	245	94	44	213
060	F	58	6.3	0.01	42	168	24	10	143
061	M	80	13.5	0.01	13	270	182	15	224
062	F	79	24.3	0.01	33	281	188	17	243
063	M	70	2.0	0.02	11	163	192	21	141
064	M	52	4.8	0.02	14	137	177	25	206
065	F	65	7.0	0.01	46	184	81	14	164
066	M	63	4.3	0.01	17	202	86	31	176
067	M	68	5.0	0.05	52	135	64	29	227
068	F	76	8.5	0.01	20	147	143	33	133
069	M	53	3.5	0.01	21	179	207	12	155
070	M	79	6.8	0.01	33	256	280	40	220

Table S5. Risk factors for the three comparison groups (yes: 1/no: 0)

Group	NO.	Dyslipidemia (1/0)	Hypertension (1/0)	Diabetes mellitus (1/0)	Smoking (1/0)	Stroke/TIA (1/0)	Family history of CAD (1/0)	Coronary artery bypass (1/0)
HC	001	0	1	-	0	-	0	-
	002	1	0	-	0	-	0	-
	003	0	1	-	1	-	0	-
	004	1	0	-	0	-	1	-
	005	1	0	-	1	-	0	-
	006	0	0	-	1	-	0	-
	007	0	1	-	1	-	0	-
	008	0	1	-	0	-	1	-
	009	0	0	-	1	-	0	-
	010	0	1	-	0	-	0	-
	011	0	1	-	0	-	0	-
	012	0	0	-	0	-	0	-
	013	0	1	-	0	-	0	-
	014	0	1	-	0	-	0	-
	015	0	1	-	0	-	0	-
	016	0	1	0	0	0	0	0

CSA	017	1	0	1	1	0	0	0
	018	0	1	1	0	0	0	1
	019	1	1	0	1	1	1	0
	020	1	0	0	0	0	0	0
	021	0	1	0	0	0	1	0
	022	1	0	0	1	0	0	1
	023	0	1	1	0	0	1	0
	024	0	0	0	0	0	0	0
	025	0	0	1	1	0	1	0
	026	0	1	0	1	0	0	0
	027	0	1	0	0	1	0	0
	028	0	1	1	0	0	0	0
	029	1	1	1	0	0	0	0
	030	0	1	0	0	0	0	0
	031	0	1	0	1	0	0	0
	032	0	1	1	0	0	0	0
	033	0	1	0	0	1	1	0
	034	1	0	1	1	0	0	0

UA	035	0	1	1	0	0	1	1
	036	1	1	1	1	0	1	0
	037	1	0	0	1	0	0	0
	038	1	1	0	1	0	0	1
	039	0	1	1	1	1	0	0
	040	0	1	1	0	0	1	0
	041	1	0	0	1	0	0	0
	042	0	1	1	0	0	0	0
	043	1	1	0	0	0	1	1
	044	0	0	0	0	0	0	0
	045	1	0	1	1	0	0	0
	046	0	1	0	0	1	1	0
	047	1	1	1	1	0	0	0
	048	0	1	0	0	0	0	0
	049	0	0	1	0	0	1	1
	050	1	1	0	0	0	0	0
	051	0	1	1	1	1	0	0
	052	0	1	1	0	0	1	0

053	1	0	0	0	0	0	0
054	1	1	0	0	0	0	0
055	0	1	1	0	0	1	0
056	0	0	1	1	1	1	0
057	0	1	1	0	0	0	0
058	0	1	0	0	0	0	1
059	0	1	1	0	0	0	0
060	1	1	0	1	1	0	0
061	0	1	0	0	0	0	0
062	0	1	0	0	0	0	0
063	0	1	0	0	0	0	0
064	0	1	0	1	0	0	1
065	0	1	0	0	0	0	0
066	0	0	0	0	0	0	0
067	0	1	1	0	0	0	0
068	0	1	0	1	0	0	0
069	0	1	0	0	0	0	0
070	0	1	0	0	0	0	0

Table S6. Medication for the three comparison groups (yes: 1/no: 0)

Group	NO.	Aspirin (1/0)	Clopidogrel (1/0)	Beta-blocker (1/0)	Calcium-channel blocker (1/0)	Oral nitrate (1/0)	ACE-inhibitor/ARB (1/0)	Statin (1/0)
HC	001	0	-	-	-	-	-	-
	002	0	-	-	-	-	-	-
	003	0	-	-	-	-	-	-
	004	1	-	-	-	-	-	-
	005	0	-	-	-	-	-	-
	006	0	-	-	-	-	-	-
	007	0	-	-	-	-	-	-
	008	1	-	-	-	-	-	-
	009	0	-	-	-	-	-	-
	010	0	-	-	-	-	-	-
	011	0	-	-	-	-	-	-
	012	0	-	-	-	-	-	-
	013	0	-	-	-	-	-	-
	014	0	-	-	-	-	-	-
	015	0	-	-	-	-	-	-
	016	0	0	1	0	0	0	1

CSA	017	1	0	0	1	1	1	1
	018	0	1	0	0	0	0	1
	019	1	0	0	1	1	1	0
	020	0	0	1	0	0	0	1
	021	1	1	0	1	1	1	1
	022	0	0	0	0	0	0	0
	023	1	0	1	0	0	0	1
	024	0	1	0	1	1	1	1
	025	1	0	0	1	1	1	0
	026	0	0	1	0	0	0	1
	027	1	1	0	0	0	0	1
	028	0	0	0	0	0	1	0
	029	0	0	1	0	0	0	1
	030	1	0	0	0	0	0	1
	031	0	0	0	0	0	0	1
	032	1	0	1	0	0	0	1
	033	0	1	0	1	1	1	1
	034	1	0	1	0	0	0	1

UA	035	1	0	0	1	1	1	1
	036	0	1	0	1	1	1	1
	037	1	0	1	0	0	0	0
	038	1	0	0	0	0	1	1
	039	0	1	1	0	0	0	1
	040	1	0	0	1	1	1	1
	041	1	0	1	0	0	0	1
	042	1	0	0	1	1	1	0
	043	0	1	1	0	0	0	1
	044	1	0	0	1	1	1	1
	045	1	0	0	1	0	0	1
	046	1	0	0	0	0	1	1
	047	0	1	1	0	0	0	1
	048	1	0	0	1	1	1	0
	049	1	0	0	0	0	0	1
	050	0	1	1	0	0	1	1
	051	0	0	0	0	0	0	1
	052	1	0	0	1	1	1	1

053	1	0	0	0	0	0	1
054	1	0	1	0	0	0	1
055	0	1	1	0	0	0	1
056	1	0	0	1	1	1	1
057	1	0	1	0	0	0	1
058	1	0	0	1	0	1	1
059	0	1	1	0	0	0	0
060	0	1	1	0	0	0	1
061	1	0	0	1	1	1	1
062	1	0	1	0	0	0	1
063	1	0	0	1	1	1	1
064	1	0	1	0	0	0	1
065	0	1	1	0	0	1	0
066	1	0	0	0	0	1	1
067	1	0	0	0	0	0	1
068	0	0	0	1	1	1	1
069	1	0	0	0	0	1	1
070	0	1	1	0	0	0	1

Table S7. TIMI UA/NSTEMI risk score for UA group (yes: 1/no: 0)

Group	NO.	Age≥65 years	≥3 risk factors for CAD	Use of ASA (last 7 days)	Known CAD (stenosis ≥50%)	>1 episode rest angina in <24 h	ST-segment deviation	Elevated cardiac markers	In Total	Risk stratification
	32	0	0	1	1	0	0	0	2	Low
	33	0	0	1	1	0	0	0	2	Low
	34	1	1	1	1	0	0	0	4	Intermediate
	35	1	1	1	1	0	0	0	4	Intermediate
	36	1	0	1	0	0	0	0	2	Low
	37	1	1	1	1	0	0	0	4	Intermediate
	38	0	0	1	1	0	0	0	4	Low
	39	1	0	1	1	0	0	0	2	Low
	40	1	1	1	1	1	0	1	6	High
	41	1	1	1	1	0	0	0	4	Intermediate
	42	0	0	1	1	1	0	1	4	Intermediate
	43	0	0	1	1	0	0	0	2	Low
	44	1	1	1	1	0	0	0	4	Intermediate
	45	1	1	1	1	0	0	0	4	Intermediate
	46	0	0	1	1	0	0	0	2	Low
	47	0	0	1	1	0	0	0	2	Low
UA	48	1	1	1	1	0	0	0	4	Intermediate
	49	0	0	1	0	0	0	1	2	Low
	50	1	1	1	1	0	0	0	4	Intermediate
	51	0	1	1	0	1	0	1	4	Intermediate
	52	1	1	1	1	0	0	1	5	High
	53	0	0	0	1	0	0	1	2	Low

54	1	1	1	1	1	0	0	5	High
55	1	0	0	1	0	0	0	2	Low
56	0	1	1	1	1	0	0	4	Intermediate
57	0	0	1	1	0	0	0	2	Low
58	1	0	1	1	1	1	0	5	High
59	1	1	0	1	0	0	1	4	Intermediate
60	1	1	1	1	1	1	1	7	High
61	1	1	1	1	1	0	1	6	High
62	1	1	1	1	0	0	0	4	Intermediate
63	1	1	1	1	0	0	0	4	Intermediate
64	0	1	0	1	0	0	0	2	Low
65	0	1	1	1	0	1	0	4	Intermediate
66	0	0	1	1	0	0	0	4	Low
67	1	1	1	1	0	1	1	6	High
68	1	1	1	1	1	0	1	6	High
69	1	1	1	1	0	1	1	6	High
70	1	1	1	1	1	1	1	7	High
